# Different Densities of Na-Ca Exchange Current in T-Tubular and Surface Membranes and Their Impact on Cellular Activity in a Model of Rat Ventricular Cardiomyocyte

**DOI:** 10.1155/2017/6343821

**Published:** 2017-02-22

**Authors:** M. Pásek, J. Šimurda, G. Christé

**Affiliations:** ^1^Institute of Thermomechanics, Branch Brno, Academy of Science of the Czech Republic, Technická 2, 61669 Brno, Czech Republic; ^2^Department of Physiology, Faculty of Medicine, Masaryk University, Kamenice 5, 62500 Brno, Czech Republic; ^3^Laboratoire de Neurocardiologie, EA4612, Université Lyon 1, 69003 Lyon, France

## Abstract

The ratio of densities of Na-Ca exchanger current (*I*_NaCa_) in the t-tubular and surface membranes (*I*_NaCa_-ratio) computed from the values of *I*_NaCa_ and membrane capacitances (*C*_m_) measured in adult rat ventricular cardiomyocytes before and after detubulation ranges between 1.7 and 25 (potentially even 40). Variations of action potential waveform and of calcium turnover within this span of the *I*_NaCa_-ratio were simulated employing previously developed model of rat ventricular cell incorporating separate description of ion transport systems in the t-tubular and surface membranes. The increase of *I*_NaCa_-ratio from 1.7 to 25 caused a prolongation of APD (duration of action potential at 90% repolarisation) by 12, 9, and 6% and an increase of peak intracellular Ca^2+^ transient by 45, 19, and 6% at 0.1, 1, and 5 Hz, respectively. The prolonged APD resulted from the increase of *I*_NaCa_ due to the exposure of a larger fraction of Na-Ca exchangers to higher Ca^2+^ transients under the t-tubular membrane. The accompanying rise of Ca^2+^ transient was a consequence of a higher Ca^2+^ load in sarcoplasmic reticulum induced by the increased Ca^2+^ cycling between the surface and t-tubular membranes. However, the reason for large differences in the *I*_NaCa_-ratio assessed from measurements in adult rat cardiomyocytes remains to be explained.

## 1. Introduction

In cardiac ventricular myocytes, the Na-Ca exchanger forms a major pathway enabling Ca^2+^ extrusion from the cells [1–3] and takes part in the process of excitation-contraction (EC) coupling [4–6]. Hence, the exchanger plays an important role in the regulation of cellular Ca^2+^ content, Ca^2+^ transient, and thereby contractility; see [7–9] for review. The majority of immunolocalization studies found the Na-Ca exchanger to be predominantly localized in the t-tubular membrane of adult ventricular cardiomyocytes [10–13]. While these studies reported the presence of Na-Ca exchanger proteins, they were not able to evaluate their activity in the t-tubular and surface membrane pools.

The recent method based on detubulation of cardiac cells [14] made it possible to assess the proportion of ionic current components and the proportion of membrane capacitances located at the t-tubular and surface membranes. Since the Na-Ca exchanger is electrogenic, this method allowed evaluating the membrane distribution of its activity. Application of this method to adult rat cardiomyocytes showed that the density of Na-Ca exchanger current (*I*_NaCa_) is higher in the t-tubular membrane [15–18]. However, the ratio of Na-Ca current density in t-tubular to surface membrane (*I*_NaCa_-ratio for short) assessed from data reported in these studies ranges between 1.7 (derived from [16]) and 25 (the lower estimate based on the data measured by Gadeberg et al. [15]). The aim of this paper is to explore the physiological consequences of such different values of the *I*_NaCa_-ratio and to discuss the possible factors that may contribute to the dispersion of these estimations.

## 2. Methods

### 2.1. Assessment of *I*_NaCa_-Ratio

The assessment of the ratio of Na-Ca current density in t-tubular membrane to that in surface membrane from experimentally measured values of *I*_NaCa_ and membrane capacitances (*C*_m_) before and after detubulation was done using the following set of relations:

(1) INaCa,intact=INaCa,sCm,s+INaCa,tCm,tCm,s+Cm,t,INaCa,detub=INaCa,sCm,s+INaCa,tCm,tft,resCm,s+Cm,tft,res,Cm,intact=Cm,s+Cm,t,Cm,detub=Cm,s+Cm,tft,res,

where *I*_NaCa,intact_, *I*_NaCa,detub_, *C*_m,intact_, *C*_m,detub_ (measured quantities) stand for the average densities of the Na-Ca exchange current (pA/pF) and the membrane capacitances (pF) in intact and detubulated cells and *I*_NaCa,s_, *I*_NaCa,t_, *C*_m,s_, *C*_m,t_ (unknown quantities) stand for the densities of the Na-Ca exchange current and membrane capacitances at the surface and t-tubular membranes. The symbol *f*_t,res_ denotes the fraction of t-tubules that resisted detubulation as estimated from confocal images of intact and detubulated myocytes.

Solution of the set of equations (1) for the unknown quantities *C*_m,t_, *C*_m,s_, *I*_NaCa,t_, and *I*_NaCa,s_ yields:

(2) INaCa,t=INaCa,intactCm,intact−INaCa,detubCm,detubCm,intact−Cm,detub,INaCa,s=INaCa,intactCm,intactft,res−INaCa,detubCm,detubCm,intactft,res−Cm,detub,Cm,t=Cm,intact−Cm,detub1−ft,res,Cm,s=Cm,detub−Cm,intactft,res1−ft,res.

Hence, the *I*_NaCa_-ratio can be expressed as

(3) INaCa,tINaCa,s=INaCa,intactCm,intact−INaCa,detubCm,detubINaCa,intactCm,intactft,res−INaCa,detubCm,detub·Cm,intactft,res−Cm,detubCm,intact−Cm,detub.

### 2.2. Model Evaluation of the Impact of *I*_NaCa_-Ratio on Cellular Activity

To evaluate the impact of the *I*_NaCa_-ratio on cellular activity, we used our model of rat ventricular cardiomyocyte [19] incorporating separate description of ion transport system in the t-tubular and surface membranes, separate dyadic, and subsarcolemmal spaces adjacent to the t-tubular and surface membranes and separate junctional sarcoplasmic reticulum (SR) compartments adjacent to the t-tubular and surface dyadic spaces (for the scheme of the model, see Figure  1 in [19]). The distribution of ion transporters between the t-tubular and surface membranes in the model is determined by the fractions of particular transporters in the t-tubular membrane (Table 1). The t-tubular fraction of Na-Ca exchangers is specified by the parameter *f*_NaCa,t_. To explore the responses of the model to different values of *I*_NaCa_-ratio, as resulted from (3) after insertion of experimental data, we set the values of *f*_NaCa,t_ according to the following relation:

(4) $$\begin{array}{c} f_{NaCa,t}=\frac{I_{NaCa,t}/I_{NaCa,s}}{C_{m,s}/C_{m,t}+I_{NaCa,t}/I_{NaCa,s}}. \end{array}$$

The simulations were performed using the computational software MATLAB v.7.2 (MathWorks, Natick, MA, USA). To attain a dynamic steady state at each stimulation frequency (0.1, 1, and 5 Hz), the model was paced for 600 s of equivalent cell lifetime by 1 ms pulses (the magnitude was set at twice the respective threshold value) under all specified conditions. The MATLAB code of the model is available at http://www.it.cas.cz/en/d3/l033/biophysics-cardiac-cells.

## 3. Results

### 3.1. *I*_NaCa_-Ratio in Adult Rat Cardiomyocytes

The *I*_NaCa_-ratio in adult rat ventricular cardiomyocytes was computed according to (3) using the values from different studies that are specified in Table 2. The experimental data published by Thomas et al. [16], Despa et al. [17], and Yang et al. [18] result in average ratios of 1.7, 4.3, and 6, respectively. However, the ratio computed from experimental data measured by Gadeberg et al. [15] is substantially higher and highly dependent on how the average values of *I*_NaCa,intact_ and *I*_NaCa,detub_ were assessed. If these values were assessed as the ratio of the average of *I*_NaCa_ amplitudes to the average of membrane capacitances for a given set of cells (Table 2, values denoted by asterisk), the resulting ratio is 25, as reported by Gadeberg et al. [15]. However, the same ratio, computed from the averages of current densities directly assessed in individual cells (see values in Table 2), amounts to 39.5. This suggests that the extent of the *I*_NaCa_-ratio in adult rat ventricular myocytes might range between 1.7 and nearly 40.

### 3.2. Impact of *I*_NaCa_-Ratio on Cellular Activity

To investigate the impact of the distribution of Na-Ca exchangers between the t-tubular and surface membranes on cellular electrophysiological activity, we used our quantitative model of the rat ventricular cardiomyocyte [19] with *C*_m,s_ and *C*_m,t_ of 65.02 pF and 34.98 pF, respectively. To meet the *I*_NaCa_-ratio of 1.7 that resulted from experimental data by Thomas et al. [16] and of 25 as reported by Gadeberg et al. [15], the original value of t-tubular fraction of *I*_NaCa_ transporters in the model (*f*_NaCa,t_ = 0.78 [19]) had to be changed to 0.48 and 0.93, respectively (see (4)).

**Figure 1:**
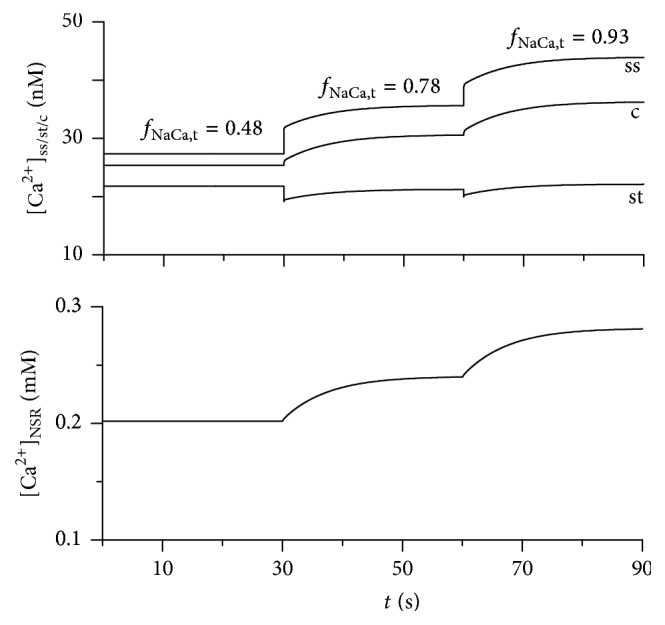
Simulation of Ca^2+^ concentration changes in the submembrane spaces (t-tubular: [Ca^2+^]_st_, surface: [Ca^2+^]_ss_), cytosol ([Ca^2+^]_c_), and network compartment of SR ([Ca^2+^]_NSR_) in unstimulated cell under different values of t-tubular fraction of Na-Ca transporters (*f*_NaCa,t_). *f*_NaCa,t_ of 0.48 and 0.93, respectively, corresponds to *I*_NaCa_-ratio of 1.7 and 25 (see Section 2.2). *f*_NaCa,t_ of 0.78 represents the original value in the model [19]. The original adjustment of membrane capacitances and extracellular ion concentrations in the model (*C*_m,s_ = 65.02 pF, *C*_m,t_ = 34.98 pF, [Na^+^]_e_ = 140 mM, [K^+^]_e_ = 5.4 mM, [Ca^2+^]_e_ = 1.2 mM) was preserved.

The effect of such changes of *f*_NaCa,t_ on resting concentrations of Ca^2+^ in subsarcolemmal spaces ([Ca^2+^]_ss_, [Ca^2+^]_st_), cytosol ([Ca^2+^]_c_), and network compartment of sarcoplasmic reticulum ([Ca^2+^]_NSR_) in unstimulated cell is illustrated in Figure 1. The increase of *f*_NaCa,t_ (that corresponds to a reduced number of Na-Ca exchangers assigned to the surface membrane) induced a rise of [Ca^2+^]_ss_ and consequently also of [Ca^2+^]_c_. This resulted in a higher Ca^2+^ load in SR. However, [Ca^2+^]_st_ was only slightly affected, because the increase of Ca^2+^ extrusion from this space, induced by higher *I*_NaCa,t_, was compensated by increased Ca^2+^ flux from the cytosol to the t-tubular subsarcolemmal space. Thus, the model showed that a redistribution of Na-Ca exchangers would lead to marked changes in net Ca^2+^ flux from the surface membrane to the t-tubular membrane through both subsarcolemmal spaces and cytosol (defined as Ca^2+^ cycling and analysed in detail in [19]).

**Figure 2:**
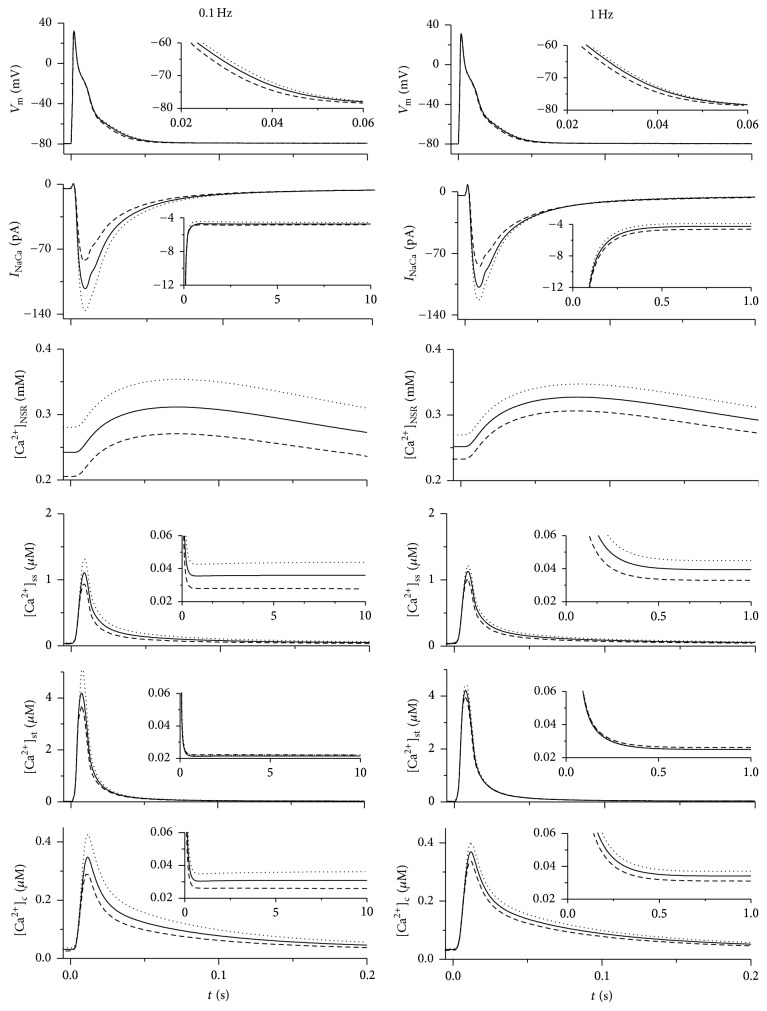
Action potential, *I*_NaCa_, and Ca^2+^ concentration changes in the network compartment of SR ([Ca^2+^]_NSR_), submembrane spaces (surface: [Ca^2+^]_ss_, t-tubular: [Ca^2+^]_st_), and the cytosol ([Ca^2+^]_c_) during 0.2 s of steady-state stimulation cycle (after 600 s) at 0.1 and 1 Hz. The full line stands for the simulations performed in the control model (*f*_NaCa,t_ set to 0.78). The dashed and dotted lines, respectively, stand for the simulations done when *f*_NaCa,t_ was set to 0.48 and 0.93 (corresponds to *I*_NaCa_-ratio of 1.7 and 25, resp.).

**Figure 3:**
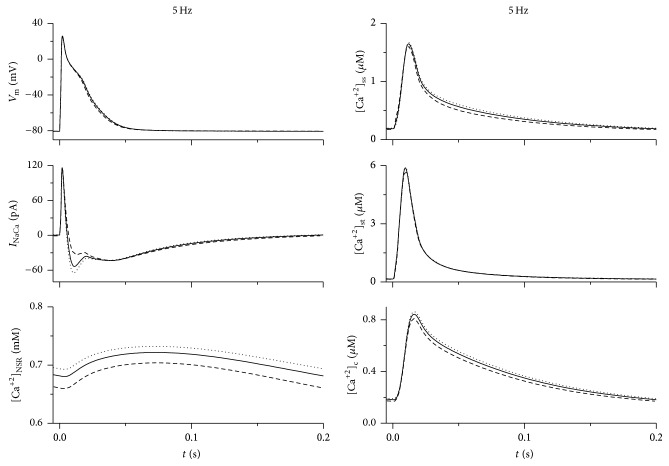
Action potential, *I*_NaCa_, and Ca^2+^ concentration changes in the network compartment of SR ([Ca^2+^]_NSR_), submembrane spaces (surface: [Ca^2+^]_ss_, t-tubular: [Ca^2+^]_st_), and the cytosol ([Ca^2+^]_c_) during 0.2 s of steady-state stimulation cycle (after 600 s) at 5 Hz (the physiological stimulation frequency corresponding to resting heart rate in rat). For further details, see legend of Figure 2.

To further explore the impact of changing *f*_NaCa,t_ on cellular electrophysiological activity, we used the model to simulate action potentials, membrane currents, and dynamic changes of Ca^2+^ concentration in individual intracellular compartments at *f*_NaCa,t_ set to 0.78, 0.48, and 0.93 at stimulation frequencies of 0.1, 1, and 5 Hz. The results obtained at 0.1 and 1 Hz (stimulation frequencies that are used in experimental works) and 5 Hz (stimulation frequency corresponding to resting heart rate in rats) are illustrated in Figures 2 and 3.

**Figure 4:**
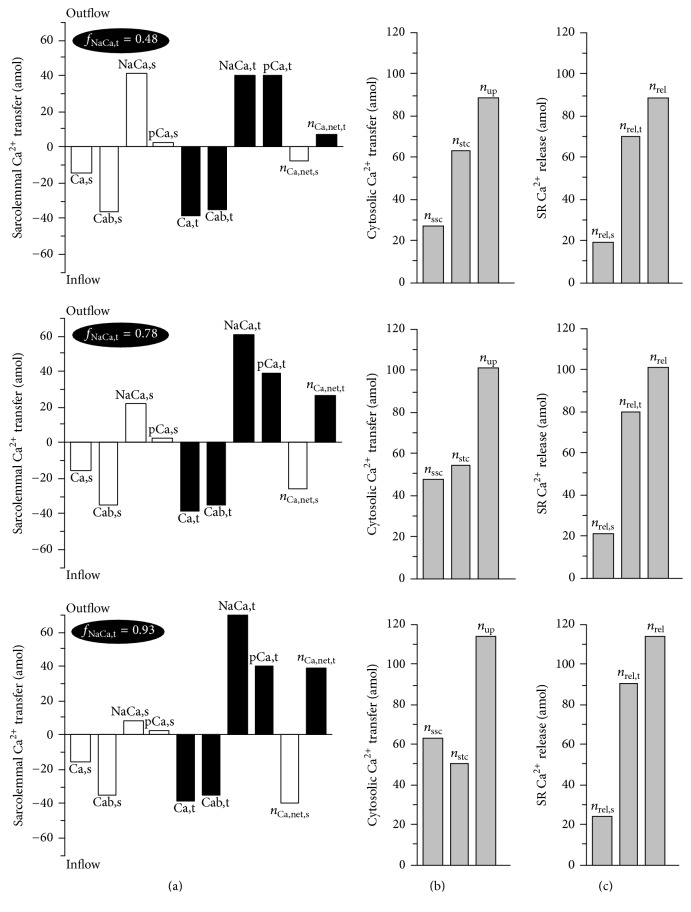
Ca^2+^ transferred by individual ion flux pathways across the cellular membrane ((a); surface, white bars; t-tubules, black bars), through the cytosol (b), and across the junctional SR membrane (c) during one steady-state cycle (1 Hz) at different values of *f*_NaCa,t_ (0.48, 0.78, and 0.93). The columns marked Ca,s, Cab,s, NaCa,s, pCa,s and Ca,t, Cab,t, NaCa,t, pCa,t show the amounts of Ca^2+^ (in amol) transferred by L-type Ca current (*n*_Ca,s_, *n*_Ca,t_), background Ca current (*n*_Cab,s_, *n*_Cab,t_), Na-Ca exchange current (*n*_NaCa,s_, *n*_NaCa,t_), and Ca pump current (*n*_pCa,s_, *n*_pCa,t_) across the surface and t-tubular membrane, respectively. The columns marked *n*_Ca,net,s_ and *n*_Ca,net,t_ represent the related net amounts of Ca^2+^ transferred across each membrane separately. The total sarcolemmal Ca^2+^ entry during the cycle equals the total extrusion and is similar at all three values of *f*_NaCa,t_ (~124 amol). The grey columns denote the amounts of Ca^2+^ that enter cytosol through the surface and t-tubular subsarcolemmal spaces (*n*_ssc_, *n*_stc_), that are sequestered from the cytosol by SR (*n*_up_), and that are released from the junctional pools of SR at the surface and t-tubular membranes (*n*_rel,s_, *n*_rel,t_, in total *n*_rel_). At any steady state, *n*_up_ = *n*_ssc_ + *n*_stc_ = *n*_rel_ = *n*_rel,s_ + *n*_rel,t_.

A redistribution of Na-Ca exchange proteins caused rather smaller but still apparent frequency-dependent change of action potential (AP). A decrease of *f*_NaCa,t_ from 0.78 to 0.48 led to a shortening of APD_90_ (duration of AP at 90% repolarisation) by approximately 7, 4, and 4% at 0.1, 1, and 5 Hz whereas increasing *f*_NaCa,t_ to 0.93 resulted in a prolongation of APD_90_ by 5, 4, and 2%, respectively. However, marked changes were evident in the level of [Ca^2+^]_NSR_ and in the peak value of transient changes of [Ca^2+^]_c_. A decrease of *f*_NaCa,t_ to 0.48 led to a reduction of [Ca^2+^]_NSR_ at the end of the cycle by 13, 7, and 3% and to a decrease of peak [Ca^2+^]_c_ by 15, 8, and 4% at 0.1, 1, and 5 Hz, respectively. Conversely, an increase of *f*_NaCa,t_ to 0.93 caused a rise of [Ca^2+^]_NSR_ by 14, 7, and 2% at the end of the cycle and an increase of peak [Ca^2+^]_c_ by 23, 9, and 2%, respectively. To clarify the mechanisms underlying the described changes induced by redistribution of Na-Ca exchangers, the amounts of Ca^2+^ moving across sarcolemma, through cytosol, and across SR membrane within a steady-state cycle at 1 Hz stimulation are shown in Figure 4 (see Discussion for details).

These data show that a redistribution of Na-Ca exchangers causes relative changes in [Ca^2+^]_NSR_ that decrease with the increase of stimulation rate (reflecting a decrease of relative changes of SR Ca^2+^ uptake at shorter stimulation cycle). This resulted in a reduction of the effect on [Ca^2+^]_c_ at higher stimulation frequencies; the increase of *f*_NaCa,t_ from 0.48 to 0.93 caused an increase of peak [Ca^2+^]_c_ by 45, 19, and 6% at 0.1, 1, and 5 Hz, respectively. Hence, the model indicates that the changes in SR Ca^2+^ load, cytosolic Ca^2+^ transient, and AP configuration caused by redistribution of Na-Ca exchangers between the t-tubular and surface membranes are potentially important in the whole range of the simulated frequencies explored.

## 4. Discussion

The assessment of the *I*_NaCa_-ratio in adult rat ventricular myocytes from electrophysiological data obtained in intact and detubulated cells [15–18] results in values ranging from 1.7 to nearly 40. The simulations on our model of rat ventricular cardiomyocytes incorporating separate description of ion transport in the t-tubular and surface membranes showed that such marked differences in the *I*_NaCa_-ratio would have important consequences for intracellular Ca^+^ cycling, Ca^2+^ transient, and thereby inotropic state of cardiomyocytes.

### 4.1. Effect of *I*_NaCa_-Ratio on Action Potential and Intracellular Ca^2+^ Transient

In 2014, we have shown that a change in the distribution of Na-Ca exchanger and other Ca^2+^ removal proteins (SERCA and sarcolemmal Ca ATPase) between membrane parts adjacent to dyadic and extradyadic spaces alters the amount of Ca^2+^ removed by each pathway from the cytoplasm and causes complex changes in intracellular Ca^2+^ dynamics and cellular Ca^2+^ cycling [23]. Despite that, a single relocation of Na-Ca exchangers (30%) from extradyadic to dyadic parts of membrane, explored at 5 Hz stimulation, had relatively small effects on Ca^2+^ transient in the cytosol (increase by ~3% [23]) due to the feedback effect of these changes on other Ca^2+^ transporting pathways.

The present study shows that changes in the distribution of Na-Ca exchanger between the t-tubular and surface membranes have substantially higher effects on cellular activity. An increase of *f*_NaCa,t_ from 0.48 to 0.93 caused a prolongation of APD_90_ by 12, 9, and 6% and a rise of steady-state cytosolic Ca^2+^ transient by 45, 19, and 6% at 0.1, 1, and 5 Hz, respectively. Thus, although this effect decreased at higher stimulation frequencies, it was still evident even at 5 Hz.

The analysis of simulated results showed that the observed changes in APD_90_ induced by different values of *f*_NaCa,t_ were caused predominantly by the related changes in *I*_NaCa_ (Figures 2 and 3). The higher the fraction of Na-Ca exchangers located in the t-tubular membrane, the higher *I*_NaCa_ (negative component) that was induced during AP due to the larger transient changes of Ca^2+^ concentration under the t-tubular membrane (compare the peak values of [Ca^2+^]_st_ and [Ca^2+^]_ss_ in Figures 2 and 3). This explains the prolongation of APD_90_ with the increase of *f*_NaCa,t_.

The increased *I*_NaCa_ extrusion of Ca^2+^ from the cell during AP would be expected to decrease the Ca^2+^ load in SR. Surprisingly, the simulations indicated that both quantities were increased or decreased simultaneously (Figures 2 and 3). To explain this apparent discrepancy, the Ca^2+^ turnover during stimulation cycle was explored in detail. As demonstrated in Figure 4, the increase in *f*_NaCa,t_ was accompanied by a decrease in the amount of Ca^2+^ extruded from the cell across the surface membrane (see the columns NaCa,s), which increased [Ca^2+^]_ss_ (see insets in Figure 2). The related increase of Ca^2+^ diffusion from the surface subsarcolemmal space into the cytosol (see *n*_ssc_ in Figure 4(b)) promoted a higher Ca^2+^ uptake by the network SR (*n*_up_) and an equivalent increase of Ca^2+^ release from junctional SR (*n*_rel_). Note, however, that because of higher fraction of junctional SR at the t-tubules (0.8 [19]), the increase of Ca^2+^ release from SR was higher at its t-tubular side (see the differences in the rise of *n*_rel,t_ and *n*_rel,s_ with *f*_NaCa,t_ in Figure 4(c)). Simultaneously, the amount of Ca^2+^ cycling from the surface to t-tubular membrane increased (see the equal rise of *n*_Ca,net,s_ and *n*_Ca,net,t_ with *f*_NaCa,t_ in Figure 4), which, together with higher *n*_rel,t_, prevented the reduction of [Ca^2+^]_st_ (see insets in Figure 2) due to the increased Ca^2+^ extrusion through the t-tubular membrane. Consequently, the increase of Ca^2+^ diffusion from the surface subsarcolemmal space into the cytosol (*n*_ssc_ = *n*_rel,s_ + |*n*_Ca,net,s_|) with *f*_NaCa,t_ was not fully compensated by the decrease of Ca^2+^ diffusion from the t-tubular subsarcolemmal space into the cytosol (*n*_stc_ = *n*_rel,t_ − *n*_Ca,net,t_). Thus, the model shows that the relocation of Na-Ca exchangers from the surface to the t-tubular membrane changes the intracellular gradients of Ca^2+^ concentration (mainly because of the rise of [Ca^2+^]_ss_), which results in an increase of Ca^2+^ entering the cytosol and SR.

It is worth mentioning that a change of *f*_NaCa,t_ affected only minutely the total Ca^2+^ transfers through individual Ca transporters in the model (*n*_Ca,s_ + *n*_Ca,t_, *n*_NaCa,s_ + *n*_NaCa,t_, *n*_pCa,s_ + *n*_pCa,t_, and *n*_Cab,s_ + *n*_Cab,t_ are nearly identical at different values of *f*_NaCa,t_ in Figure 4). The increased Ca^2+^ extrusion via *I*_NaCa_ during AP at higher values of *f*_NaCa,t_ was compensated by its reduction in the later phase of the stimulation cycle (see insets in Figure 2) due to lower [Ca^2+^]_st_ (compared with [Ca^2+^]_ss_). This implicated that the total transsarcolemmal Ca^2+^ transfer was nearly unaffected by higher *f*_NaCa,t_. Nevertheless, due to the increased Ca^2+^ cycling, an increased fraction of Na-Ca exchangers at the t-tubules may have a sizeable positive inotropic effect and may play an important role in the adaptation of the heart to the increase in hemodynamic demand during development. Besides, the inverse process could, at least partly, explain the relation between the reduction of cytosolic Ca^2+^ transient observed at 1 Hz stimulation in cells from failing hearts [21] and a decrease of *I*_NaCa_-ratio in the same cells [15]. The frequency dependence of this effect, as revealed by the model, reflected a decrease in relative changes of SR Ca^2+^ uptake at shorter stimulation cycle; when *f*_NaCa,t_ was increased from 0.48 to 0.93, the amount of Ca^2+^ entering SR during a stimulation cycle increased by 69 and 28% at 0.1 and 1 Hz (see the increase in *n*_up_ in Figure 4) and by 6% at 5 Hz.

To sum up, changes in the *I*_NaCa_-ratio affect more the intracellular Ca^2+^ turnover and related excitation-contraction coupling than the electrical activity of rat ventricular cell in our model. However, the reason of sizeable differences in the *I*_NaCa_-ratio as evaluated in cardiomyocytes from adult healthy rats remains to be explained.

### 4.2. Which Factors Might Be Responsible for Different *I*_NaCa_-Ratio in Rat Cardiomyocytes?

The question about the factors affecting the *I*_NaCa_-ratio in rat ventricular cardiomyocytes deserves attention. The basic characteristics of rats used by Gadeberg et al. [15], Thomas et al. [16], Despa et al. [17], and Yang et al. [18] are summarised in Table 3. As follows from the table, the largest differences between the animals appear to be in the age of rats. The rats used by Gadeberg et al. [15] were more than twice older than those used in all other studies. The studies published by Dan et al. [10] and Chen et al. [12] clearly showed that the distribution of Na-Ca exchangers in rabbit ventricular cardiomyocytes changes during development. While less than 10% of Na-Ca exchange proteins were present at the cell interior (t-tubular membrane) in myocytes from newborn rabbits, about 67% of these proteins were located there at their maturity (8 weeks postpartum) [10]. This distribution of Na-Ca exchangers results in an *I*_NaCa_-ratio of 3.7 in our rat model, which is slightly smaller than the average ratio of 4.3 computed from data by Despa et al. [17] obtained from approximately eleven-week-old rats. However, the rats used by Thomas et al. [16], although being older than those used by Yang et al. [18], exhibited a smaller ratio (1.7 versus 6). This inconsistency in age-dependency of experimental data suggests that the *I*_NaCa_-ratio in adult rat ventricular cardiomyocytes is likely affected by other factors. Besides the age of animals, a potentially important role may also be assigned to different experimental conditions and precision in measurement/evaluation of experimental data. The need to explain this physiologically important point strongly calls for new electrophysiological and immunolabelling data from adult rats of different ages.

## 5. Conclusion

The *I*_NaCa_-ratio in adult rat ventricular cells, as assessed from electrophysiological data published to date, yields strikingly different values. The simulations performed on a model of rat cardiomyocyte showed that such differences in the *I*_NaCa_-ratio would significantly affect the intracellular gradients of Ca^2+^ concentration, SR Ca^2+^ load, and thus the cellular inotropic state. The reason for these differences is however unclear. More studies focused on location and precise assessment of the fraction of Na-Ca exchangers within t-tubules would be of considerable value and would help to explain the differences resulting from available experimental data.

## Figures and Tables

**Table 1:** Fractions of ion transporters in the t-tubular membrane of rat ventricular cardiomyocytes as estimated from experimental measurements of ionic currents and membrane capacitances in intact and detubulated cells. The individual fractions *f*_X,t_ relate to ion transporters mediating: fast Na current (*I*_Na_), L-type Ca current (*I*_Ca_), transient outward K current (*I*_Kto_), steady-state outward K current (*I*_Kss_), inward rectifying K current (*I*_K1_), hyperpolarization-activated current (*I*_f_), background currents (*I*_Kb_, *I*_Nab_, *I*_Cab_), Na-Ca exchange current (*I*_NaCa_), Na-K pump current (*I*_NaK_), and Ca pump current (*I*_pCa_). Adopted from supplement to [19].

*f*_Na,t_	0.38
*f*_Ca,t_	0.8
*f*_Kto,t_	0.46
*f*_Kss,t_	0.86
*f*_K1,t_	0.47
*f*_f,t_	0.49
*f*_Nab,t_	0.49
*f*_Cab,t_	0.49
*f*_Kb,t_	0.49
***f***_**NaCa**,**t**_	**0.78**
*f*_NaK,t_	0.64
*f*_pCa,t_	0.95

**Table 2:** Membrane capacitance and *I*_NaCa_ assessed in intact and detubulated cells by Gadeberg et al. [15], Thomas et al. [16], Despa et al. [17], and Yang et al. [18]. The numerical values of *C*_detub_, *I*_NaCa,intact_, and *I*_NaCa,detub_ related to the paper published by Gadeberg et al. [15] were kindly provided by Professor Clive Orchard from University of Bristol. The data represent average values ±SE and related number of cells (*n*). The data denoted by asterisk represent current densities computed from ratios of average values of *I*_NaCa_ amplitudes (*I*_NaCa,intact_ = −55.17 pA, *I*_NaCa,detub_ = −16.4 pA) and membrane capacitances in intact and detubulated cells. The values of *f*_t,res_ denoted by black-filled circles were quantified additionally [20].

*C*_intact_ (pF)	*C*_detub_ (pF)	*I*_NaCa,intact_ (pA/pF)	*I*_NaCa,detub_ (pA/pF)	*f*_t,res_	ref.
240.2 ± 21 *n* = 12	206.2 ± 11 *n* = 9	−0.266 ± 0.08 *n* = 12 −0.23^*∗*^	−0.078 ± 0.02 *n* = 9 −0.08^*∗*^	0.16	[15]

204 ± 11 *n* = 23	150 ± 7 *n* = 13	2.5 ± 0.2 *n* = 23	2.1 ± 0.1 *n* = 13	0	[16]

156 ± 7 *n* = 24	106 ± 5 *n* = 19	0.19 ± 0.03 *n* = 12	0.1 ± 0.03 *n* = 11	0.08^∙^	[17]

193 ± 41 *n* = 25	143 ± 34 *n* = 25	0.05 ± 0.01 *n* = 9	0.024 ± 0.006 *n* = 8	0.08^∙^	[18]

**Table 3:** Basic characteristics of rats used in so far published studies dealing with the distribution of Na-Ca exchangers between the t-tubular and surface membranes. The strain, gender, and weight of rats used by Thomas et al. [16] and Yang et al. [18] were kindly provided by Professor Ivar Sjaastad from University of Oslo and by Professor Clive Orchard from University of Bristol. The values denoted by asterisk were obtained from the growth chart at http://www.criver.com/.

Strain	Gender	Age (weeks)	Weight (g)	Ref.
Wistar	Male	~25	~460 g	[15, 21]
Wistar	Male	~9^*∗*^	~300 g	[16]
Sprague-Dawley	Male	~11^*∗*^	~300 g	[17, 22]
Wistar	Male	~7^*∗*^	~250 g	[18]
